# 16S rRNA and Omp31 Gene Based Molecular Characterization of Field Strains of *B. melitensis* from Aborted Foetus of Goats in India

**DOI:** 10.1155/2013/160376

**Published:** 2013-12-18

**Authors:** Ajay Singh, Vivek Kumar Gupta, Amit Kumar, Vikas Kumar Singh, Shivasharanappa Nayakwadi

**Affiliations:** ^1^College of Biotechnology, Uttar Pradesh Pandit Deen Dayal Upadhayay Pashu Chikitsa Vigyan Vishwavidyalaya Evum Go-Anusandhan Sansthan (DUVASU), Mathura 281001, India; ^2^Animal Health Division, Central Institute for Research on Goats (CIRG), Makhdoom, Mathura 281001, India; ^3^Department of Veterinary Microbiology, Uttar Pradesh Pandit Deen Dayal Upadhayay Pashu Chikitsa Vigyan Vishwavidyalaya Evum Go-Anusandhan Sansthan (DUVASU), Mathura 281001, India

## Abstract

Brucellosis is a reemerging infectious zoonotic disease of worldwide importance. In human, it is mainly caused by *Brucella melitensis*, a natural pathogen for goats. In India, a large number of goats are reared in semi-intensive to intensive system within the close vicinity of human being. At present, there is no vaccination and control strategy for caprine brucellosis in the country. Thus, to formulate an effective control strategy, the status of etiological agent is essential. To cope up with these, the present study was conducted to isolate and identify the prevalent *Brucella* species in caprine brucellosis in India. The 30 samples (fetal membrane, fetal stomach content and vaginal swabs) collected throughout India from the aborted fetus of goats revealed the isolation of 05 isolates all belonging to *Brucella melitensis* biovars 3. All the isolates produced amplification products of 1412 and 720 bp in polymerase chain reaction with genus and species specific 16S rRNA and omp31 gene based primers, respectively. Moreover, the amplification of omp31 gene in all the isolates confirmed the presence of immuno dominant outer membrane protein (31 kDa omp) in all the field isolates of *B. melitensis* in aborted foetus of goats in India. These findings can support the development of omp31 based specific serodiagnostic test as well as vaccine for the control of caprine brucellosis in India.

## 1. Introduction

Brucellosis is an infectious zoonotic disease of worldwide importance in both animals and humans [1, 2] caused by microorganisms belonging to the genus *Brucella*, Gram-negative facultative intracellular bacteria [3–5]. It is a bacterial zoonosis of worldwide importance, and of major public health and economic significance [4, 6, 7]. There are few different species of *Brucella*, each with slightly different host specificity. Six species of *Brucella* have been identified: *B. melitensis*,* B. suis*,* B. abortus*,* B. ovis*,* B. neotomae*, and* B. canis *[8]. This classification is based on the animal host specificity, susceptibility to dyes, metabolic patterns, phage typing, and serological testing [9–12]. *B. melitensis* uses the sheep and goats as its preferred natural hosts but other animals and human being may also be infected [13, 14]. Other species like *B. abortus*,* B. suis*,* B. ovis*, and *B. neotomae* mainly infect cattle, pigs, sheep, and rodents. Recently, new species were discovered: in marine mammals (*B. pinnipedialis *and *B. cetacea*), in the common vole *Microtus tusarvalis* (*B. microti*), and even in a breast implant (*B. inopinata*) [2].

Caprine brucellosis causes serious economic losses by way of abortions and stillbirths, besides being potentially hazardous to the animal handlers. Infected parturitions (normal birth or abortion) and infected males play important roles in the spread of infection in herds [2, 3, 13, 14]. Control of infection is necessary not only to reduce economic of losses but also to avoid contamination in man [15]. In India, 13.4% of kids are expected to be lost due to *Brucella* originated abortions and stillbirth in semi-intensively managed goat herds [16]. Because of serious economic importance and medical consequences of brucellosis, especially in developing countries [1, 17], efforts have been made to prevent and control the disease through the use of vaccines [2, 18]. The continued improvement of vaccines against *B. melitensis* is important for the control and eradication of the disease in sheep, goats, and human beings [18–20]. For that, isolation and characterization of the existing species is not only essential but also a key to the success in the form of diagnostic test or vaccine [3, 7, 20–22]. Thus, to establish the etiological agent of caprine brucellosis and to determine the presence of biotypes of *Brucella* spp. in caprine abortion cases in India, isolation and identification of causing agent is preliminary and essential step.

As unequivocal diagnosis is by bacteriological identifications of the causative agent [23] and for the confirmation of brucellosis, isolation is still a gold standard test either for the screening of the infection or preparing eradication programs [24]. Moreover, for further confirmation of *Brucella* species, various molecular methods have been developed [25–27] and most of them are based on the detection of omp31 gene in *B. melitensis *[28]. These outer membrane proteins (Omps) have been isolated and characterized from several species of *Brucella* initially for the development of subcellular vaccines [25–29]. *Brucella abortus* strains contain two major Omps designated as omp25 (25–27 kDa) and omp2 or porin (36–38 kDa) [25, 26]. Similarly, *B. melitensis* contains two Omps with apparent molecular masses of 25–27 kDa and 31–34 kDa, now designated as omp31 [28] and 28 kDa, designated as omp28 [27]. The omp31 gene of *B. melitensis* 16 M has been cloned and expressed on the surface of *E. coli* [28] and was shown to protect mice model and natural host against a *B. ovis *challenge [30, 31]. Thus, there is an increasing interest worldwide on cloning and molecular characterization of omp31 gene from different strains of *B. melitensis* with the ultimate goal of suitable, safe, and effective vaccine and development of *B. melitensis* specific diagnostic test. Hence, the present study was planned to know the involvement of *Brucella* species and biovars with molecular characterization of omp31 gene encoding an immuno dominant outer membrane protein (31 kDa omp) from field strain of *B. melitensis* in aborted foetus of goats in India.

## 2. Materials and Methods

### 2.1. Samples

Thirty samples collected from the aborted goats and fetus just after abortion (fetal membrane, fetal stomach content, and vaginal swabs) aseptically were subjected to isolation of bacteria and its molecular characterization through PCR.

### 2.2. Isolation and Identification of *Brucella*

For the isolation of *Brucella*, material from different sources was inoculated on sterile plates of Brucella selective agar media with hemin and vitamin k_1_ media (Hi Media) and incubated at 37°C for 48 h. The plates were observed at every 24 h for the development of growth. After the growth, the colonies suspected for *Brucella* on the basis cultural characteristics [23] were picked up and streaked to another Brucella selective agar with hemin and vitamin k_1_ plates and incubated at 37°C for 2 days to obtain pure culture.

### 2.3. Cultural Characterization of Isolates

The pure cultures of the isolates examined by morphological examination were inoculated on Brucella selective agar medium, MacConkey Lactose agar (MLA) and Sheep blood agar [10]. The isolates showing characteristic colonies on Brucella selective agar medium, no growth on MacConkey Lactose agar (MLA) and nonhemolytic colonies on blood agar were maintained in Serum dextrose agar for further studies.

### 2.4. Morphological Characterization of Isolates

The isolates suspected for *Brucella *were subjected to Gram staining and Stamp's modified Ziehl-Neelsen (MZN) staining [23] for checking the purity of cultures and morphological characters. Stamp modified Ziehl-Neelsen staining method was performed with 0.4% basic fuchsin solution, followed by rapid decolouration with 0.5% acetic acid solution, and counterstaining with 1% methylene blue or malachite green solution. The smears were examined microscopically with an oil-immersion objective lens (×100).

### 2.5. Biochemical Confirmation of Isolates

Pure suspected *Brucella* isolates, maintained in Serum dextrose agar, were analysed for their biochemical profiles for the differentiation of *Brucella* species on the basis of biochemical tests, namely, catalase, oxidase, urea hydrolysis, nitrate reduction tests, indole production, citrate utilization, methyl red and voges-proskauer tests as per the standard methods [23, 32].

### 2.6. Biotyping of *Brucella* Isolates

Cultures showing typical *Brucella* characteristics were subjected to biotyping techniques such as H_2_S production, growth in the presence of thionin and basic fuchsin (10–40 *μ*g/mL) dye incorporated into Tryptic soya agar at different concentrations (1 : 25,000, 1 : 50,000, and 1 : 100,000) from 0.1% stock solution (with distilled water), and CO_2_ requirement immediately after the primary isolation as well-described method [33]. Lead acetate strips were used to identify the production of H_2_S during growth, and the growth test on media containing streptomycin (2.5 *μ*g/mL) was performed to discriminate the isolates from vaccine strain Rev1 as per standard procedures [11, 12].

### 2.7. Molecular Characterization of *Brucella melitensis* Isolates

For molecular confirmation of these isolates, amplification of 16S rRNA and omp31 genes was performed by using Taq PCR master mix kit (Qiagen).

#### 2.7.1. Extraction of DNA from Colonies

The isolate colonies from Serum dextrose agar were transferred on Brucella selective agar with hemin and vitamin k_1_ plates. Then, few colonies were picked and transferred into 2 mL eppendorf tube containing 1 mL of sterile PBS (pH: 7.4). The suspension in PBS was centrifuged at 10,000 rpm for 10 min at 10°C. The supernatant was discarded and the pellet was used for extraction of DNA. Deoxyribonucleic acid (DNA) was isolated by using mdi kit (Advanced micro device Pvt. Ltd., India).

#### 2.7.2. Polymerase Chain Reaction

DNA isolated from bacterial isolate colonies was used for polymerase chain reaction for the amplification of 16S rRNA and omp31 genes for the confirmatory identification of *Brucella melitensis* by using Taq PCR master mix kit (Qiagen). 16S rRNA gene is specific to the genus *Brucella* while the omp31 is a species specific gene to the *Brucella melitensis* [34, 35]. For the amplification of 16S rRNA gene primers, earlier described forward primer (5′-AGAGTTTGATCCTGGCTCAG-3′) and backward primer (5′-ACGGCTACCTTGTTACGACTT-3′) were used [36]. Similarly, for the amplification of species specific omp31 gene, a set of forward (5′-TGACAGACTTTTTCGCCGAA-3′) and backward (5′-TATGGATTGCAGCACCG-3′) primers were applied [28]. The 25 *μ*L of PCR reaction was prepared with 12.5 *μ*L Taq PCR master mix (2x); 1 *μ*L forward primer (10 pmol/*μ*L); 1 *μ*L reverse primer (10 pmol/*μ*L); 2 *μ*L template DNA, and 8.5 *μ*L nuclease free water. The final reaction volume of 25 *μ*L for each sample was used in thermal cycler (Techne, TC 4000). The amplification of 16S rRNA gene was conducted with initial denaturation at 95°C for 5 min, denaturation at 95°C for 30 sec, annealing at 54°C for 1.5 min, extension at 72°C, 1.5 min, and finally the final extension at 72°C for 10 min. The omp31 gene amplification was performed with initial denaturation at 95°C for 5 min, denaturation at 95°C for 1 min, annealing at 58°C for 1 min, extension at 72°C for 1 min, and finally the final extension at 72°C for 10 min.

#### 2.7.3. Quantitation and Quality Assessment of DNA of PCR Products by Agarose Gel Electrophoresis

For the electrophoresis of PCR products, 1% agarose gel was prepared in TAE buffer (Bangalore Genei). Ethidium bromide (10 mg/mL) was added to final concentration of 0.5 *μ*g/mL and mixed gently prior to casting of gel. The PCR product (8 *μ*L) was mixed with 2 *μ*L of loading dye in gel apparatus (GeNei, India) and run at 70–80 volt/cm for 40–50 min till the dye reached the half of the gel. The gel was photographed under the UV illuminator (Alpha Innotech). The size of the amplicon was assessed on the basis of comigration of standard DNA ladder of molecular weight in the range of 100–1000 bp and 1000–2000 bp for the amplifications of 16S rRNA and omp31 genes, respectively (Banglore Genei).

## 3. Results

All the aborted materials collected from the cases of abortions were inoculated on Brucella selective agar plates and the isolates producing characteristic, very small, glistening and smooth, round, and pin-point colonies were further transferred on MacConkey Lactose agar (MLA) and Sheep blood agar. The isolates which did not grow on MacConkey agar (MLA) and are to be nonhemolytic on blood agar were examined for morphological characters by Gram and Modified Ziehl-Neelsen (MZN) staining. Microscopic examination of Gram-stained cultures revealed small Gram-negative coccobacilli and, on modified Ziehl-Neelsen (MZN) staining, organisms stained red against a blue background. These isolates were further assessed for the biochemical characters and the isolates were found positive for catalase, oxidase, urea hydrolysis and nitrate reduction tests and negative for indole production, citrate utilization, and methyl red, and voges-proskauer tests were suggestive of *Brucella *species (Table 1).

**Figure 1:**
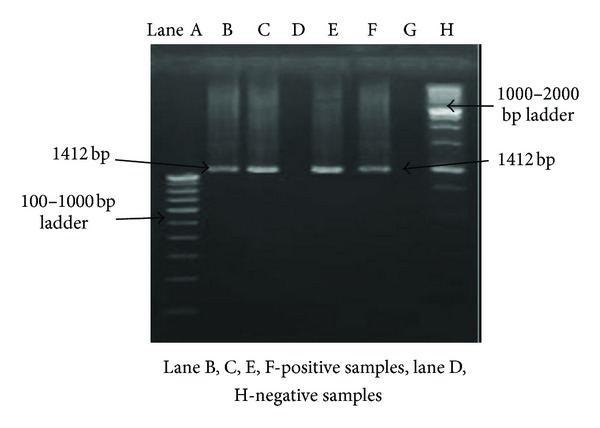
Amplification of genus *Brucella* specific 16S rRNA gene.

**Figure 2:**
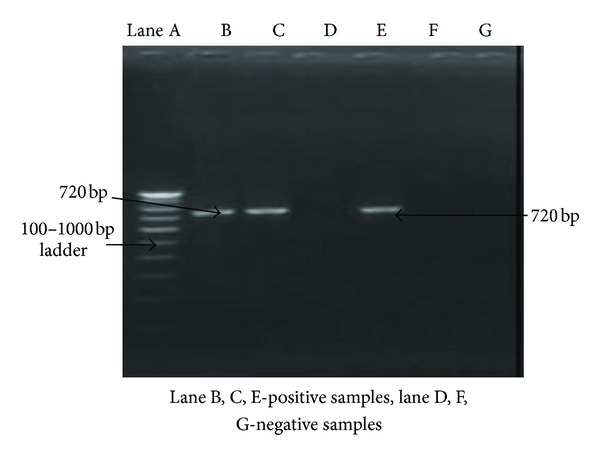
Amplification of *Brucella melitensis* species specific omp31 gene.

Thus, on the basis of cultural, morphological, and biochemical characteristics, five isolates were identified as *Brucella* species. For the conventional diagnosis of *Brucella *species, all the isolates were differentiated phenotypically into species and partially to biovars using parameters such as CO_2_ requirement, H_2_S production, and growth on media plates containing thionin and basic fuchsin (10–40 *μ*g/mL) dye incorporated into Tryptic soya agar at three different concentrations (1 : 25,000, 1 : 50,000, and 1 : 100,000). The growth of all the 5 isolates on media with thionin at only 40 *μ*g/mL (1 : 25,000) concentration and basic fuchsin at all concentrations suggested these isolates as *Brucella melitensis *biovar 3 (Table 1). For the confirmation of genus and species when DNA of these isolates were subjected to 16S rRNA and omp31 gene amplification for identification and characterization, an amplified product of about 1412 bp (Figure 1) and 720 bp (Figure 2) size was found in all the 5 isolates on agarose gel electrophoresis.

## 4. Discussion 

All the 5 isolates obtained from the cases of aborted fetus were initially confirmed by the cultural, morphological, and biochemical tests as *Brucella *species [10, 23, 32]. These 5 isolates revealed the presence of *Brucella* organism on Brucella selective agar medium with the development of characteristic colonies similar to the earlier reports [10]. These findings are also in the concurrence to the reports of isolation of *Brucella melitensis* in 25 cases in the Thrace Region [37]. All the isolates revealed morphological characters similar to previous findings [23] with biochemical tests in concurrence with the findings of other studies [23, 32]. For morphological characterization Gram staining and modified Ziehl-Neelsen (MZN) staining [23] and for the differentiation of *Brucella *species on the basis of biochemical tests, different tests, namely, catalase, oxidase, urea hydrolysis, nitrate reduction tests, indole production, citrate utilization, methyl red, and voges-proskauer tests (Table 1) were applied as per the method recommended earlier [32]. Similar to the earlier reports [32], all the *Brucella* isolates were found positive for catalase, oxidase, urea hydrolysis, and nitrate reduction tests and negative for indole production, citrate utilization, methyl red, and voges-proskauer tests (Table 1) revealing them to be *Brucella *species. Thus, on the basis of cultural, morphological, and biochemical characteristics, the organisms were identified as *Brucella *species [23, 32].

The isolates were further differentiated phenotypically into species and partially to biovars using parameters such as CO_2_ requirement, H_2_S production, and growth on media plates containing thionin and basic fuchsin dyes at three different concentrations (Table 1) [11, 12, 23, 33]. Accordingly, *Brucella* species grown on Tryptic soy agar media containing both thionin and basic fuchsin dyes at concentration of 40 *μ*g/mL (1 : 25,000), 20 *μ*g/mL (1 : 50,000), and 10 *μ*g/mL (1 : 100,000) have been taken as *Brucella melitensis*, whereas isolates with no growth at all concentrations in both the cases (thionin and basic fuchsin) were considered as *Brucella melitensis *biovars 2 and those grown on media with thionin at only 40 *μ*g/mL (1 : 25,000) concentration and basic fuchsin at all concentrations have been considered as *Brucella melitensis *biovar 3 (Table 1) [11, 12, 32]. These findings suggested all the isolates as *Brucella melitensis *biovar 3 and are in agreement with the earlier reports [3, 9, 10]. However, in earlier reports [37], 25 cases of biotypes 1 and 3 of biotype 2 among 29 *B. melitensis* isolates were observed. Whereas, in about 78 *B. melitensis* isolates, collected from various parts of Turkey, 69 and 9 isolates were identified as biotype 3 and biotype 1, respectively [11, 12]. Thus, *Brucella melitensis *biovar 3 is mainly responsible for the clinical form of brucellosis in goats and leads to abortions and other clinical signs.

The molecular approaches appeared to be faster and more sensitive than traditional bacteriological tests [8, 38–40]. The 16S rRNA component of 30S small subunit of prokaryotic ribosomes contains hyper-variable regions that provide species specific signature sequences useful for bacterial identification, so 16S rRNA gene can be used as the diagnostic target in the PCR for confirmatory identification of *Brucella melitensis*. In this study, we have primarily focused on the applicability of 16S rRNA gene as a rapid confirmatory identification tool for *Brucella* genus as per the procedure adopted earlier [36]. The extracted DNA was PCR amplified using *Brucella* genus specific primers [36]. A PCR product of about 1412 bp size of 16S rRNA from all the isolates of *B. melitensis *was obtained (Figure 1). It confirmed that all the isolates belong to genus *Brucella*. The advantage of this method is that results can be obtained within 1 day as compared to 7 days by traditional microbiological testing. Previous work on other bacteria has indicated that differences in 16S rRNA gene sequences may be useful for subtyping or for the differentiation of virulent subtypes from nonvirulent subtypes [41, 42]. Low variability in the 16S rRNA locus has been noted as an impediment in using 16S rRNA gene sequencing to discriminate at the species level [43]. In recent studies of other biothreat, select agents have indicated that even subtle differences in the 16S rRNA gene sequence may be used for differentiating and identifying closely related species, which are often cross-reactive in biochemical identification systems commonly used in diagnostic laboratories [42, 44].

A multiplex system has been developed that is sensitive for *Brucella* spp. and is able to differentiate between *B. melitensis* and *B. abortus* [45]. However, discrepant results were observed with some *B. abortus *isolates. So far, none of these assays have been accepted for common use in diagnostic laboratories. Moreover, only a few studies in the literature [13, 14, 46–48], however, address direct detection of *Brucella melitensis* in clinical specimens of goat origin. In the present study, a PCR based assay for the rapid and specific laboratory diagnosis of *Brucella melitensis* directly from tissue and blood using specific primers for the PCR amplification of a 720 bp region on the sequence encoding the 31 kDa immunogenic *B. melitensis* protein (omp31) [13, 14] was applied for the confirmation of *Brucella melitensis* from genomic DNA with species specific primers [28]. All the isolates produced an amplified product size of about 720 bp (Figure 2). Thus, all the isolates obtained from the cases of abortion in goats belong to *B. melitensis* as PCR amplification of omp31 gene (720 bp) from previously extracted genomic DNA using specific oligonucleotide primers [49] confirmed the presence of this gene in *B. melitensis* and its absence in *B. abortus* [3, 22, 50–54]. These findings are in agreement with others which reported *B. melitensis *from such cases of sheep and goat abortions [11, 12, 37]. Moreover, the amplification of omp31 gene confirms presence of immuno dominant outer membrane protein (31 kDa omp) in all the field isolates of *B. melitensis *in aborted foetus of goats in India.

## 5. Conclusions

*Brucella melitensis* is mainly responsible for the brucellosis in goats and also transmission of infection to human being. For the control of the *Brucella melitensis,* effective diagnosis and vaccination are required and all these can only be decided after epidemiological studies including isolation of etiological agents from the clinical cases to establish prevalent species and biovars. A country like India with huge goat population being reared in the close vicinity of human is always on the edge of *Brucella* zoonoses. In such scenario, the findings of the present study that *Brucella melitensis* biovars 3 are the most prevalent strain in country with well-established immuno dominant outer membrane protein (31 kDa omp) can be a milestone for the development of effective diagnostic as well as prophylactic agent to eradicate the disease.

## Figures and Tables

**Table 1:** Species and biovar differentiation of the species of the genus *Brucella* isolates.

*Brucella* isolates	Source	Growth characteristics					Monospecific sera		Phage typing						Interpretation
		Urea	H_2_S	CO_2_	BF	TH	A	M	Tb	Wb	BK_2_	Fi	Iz	R/C	
P1	Fetal membrane	++	−	−	+	+	−	+	NL	NL	CL	NL	PL	NL	*Brucella melitensis* biovar 3
P2	Stomach content	++	−	−	+	+	−	+	NL	NL	CL	NL	PL	NL	*Brucella melitensis* biovar 3
P3	Stomach content	++	−	−	+	+	+	+	NL	NL	CL	NL	PL	NL	*Brucella melitensis* biovar 3
P4	Stomach content	++	−	−	+	+	+	+	NL	NL	CL	NL	PL	NL	*Brucella melitensis* biovar 3
P5	Vaginal Swab	++	−	−	+	+	+	+	NL	NL	CL	NL	PL	NL	*Brucella melitensis* biovar 3

BF: basic fuchsin at 20 *μ*L/mL (1/50,000 w/v), TH: thionin at 20 *μ*L/mL (1/50,000 w/v), CL: confluent lysis, PL: partial lysis, NL: no lysis.
